# Differences in pregnancy outcomes and obstetric care between asylum seeking and resident women: a cross-sectional study in a German federal state, 2010–2016

**DOI:** 10.1186/s12884-018-2053-1

**Published:** 2018-10-24

**Authors:** Kayvan Bozorgmehr, Louise Biddle, Stella Preussler, Andreas Mueller, Joachim Szecsenyi

**Affiliations:** 1 Department of General Practice and Health Services Research, University Hospital Heidelberg, Marsilius Arkaden, INF 130.3, 69120 Heidelberg, Germany; 2 Institute for Medical Biometry and Informatics, University Hospital Heidelberg, Heidelberg, Germany; 3 Clinic of Gynaecology, Karlsruhe City Hospital, Karlsruhe, Germany

**Keywords:** Migration, Asylum seekers, Health inequality, Pregnancy outcome, Caesarian section, Stillbirth, Reception center, Maternity care, Postnatal care, Social epidemiology

## Abstract

**Background:**

Despite large numbers of asylum seekers, there is a lack of evidence on pregnancy outcomes and obstetric care of asylum seeking women in Germany.

**Methods:**

Cross-sectional study (2010–2016) using administrative data of the main referral hospital for pregnant asylum seekers of the reception center of a large federal state in South Germany. Inclusion criteria: women aged 12–50 years, admitted in relation to pregnancy, childbirth or post-partum complications. Outcomes: differences between asylum seekers and residents in the prevalence of high-risk pregnancy conditions, abortive outcomes/stillbirths, peri- and postnatal maternal complications, neonatal complications, and caesarean sections. Analysis: odds ratios (OR) and 95% confidence intervals (CI) obtained by single and multiple logistic regression analysis. Attributable fractions among the exposed (*Afe*) and among the total population (*Afp*) were calculated for selected outcomes.

**Results:**

Of 19,864 women admitted in relation to pregnancy, childbirth or post-partum complications, 2.9% (*n* = 569) were asylum seekers. Adjusted odds for high-risk pregnancy conditions (OR = 0.76, 95%CI: 0.63–0.91, *p* < 0.0001), caesarean sections (OR = 0.84, 95%CI 0.66–1.07, *p* = 0.17) and perinatal complications (OR = 0.65, 95%CI: 0.55–0.78, *p* < 0.0001) were lower; those for abortive outcomes/stillbirths (OR = 1.58, 95%CI: 1.11–2.20, *p* = 0.01) and postnatal complications (OR = 1.80, 95%CI: 0.93–3.19, *p* = 0.06) higher among asylum seeking women relative to residents in models adjusted for age, length of admission, and high-risk pregnancy conditions. The *Afe* for abortive outcomes and stillbirths among asylum seekers was 40.3% (95% CI, 16.3–56.5) and the *Afp* was 1.8%. The *Afe* for postnatal complications was 53.1% (95% CI, 7.1–74.0) and the *Afp* was 3.1%.

**Conclusion:**

Asylum seeking women are at higher risk of abortive outcomes/stillbirths and show a tendency towards higher postnatal complications. This excess risk calls for adequate responses by health care providers and policy makers to improve outpatient postnatal care in reception centers and mitigate adverse birth outcomes among asylum seeking women. Although further research is needed, scaling-up midwivery care, improving outreach by maternity care teams, and routinely identifying and addressing mental illness by psychosocial services could be ways forward to improve outcomes in this population.

**Electronic supplementary material:**

The online version of this article (10.1186/s12884-018-2053-1) contains supplementary material, which is available to authorized users.

## Background

Asylum seeking pregnant women are considered a vulnerable population group with special needs [1]. The Asylum Seekers’ Benefits Act (Asylbewerberleistungsgesetz), which regulates legal entitlements to healthcare for asylum seekers in Germany, grants unrestricted access to needed health care for this population [2, 3]. Despite equal legal access to health care, pregnant asylum seekers may face numerous barriers to maternity care as a result of limited availability of specialized services in reception centers as well as geographical and language barriers to accessing regular health services outside reception centers [4, 5]. Adverse living conditions and health system factors of the country of origin, negative experiences and stressors during the peri-migration phase, as well as structural factors related to reception in destination countries may also put asylum seeking pregnant women at high risk of adverse birth outcomes [6–8].

Adverse birth outcomes not only impact the mortality and morbidity of the infant, but can also have detrimental health effects throughout the child’s lifecourse, including an increased risk of coronary heart disease, diabetes and hypertension [9]. Thus, adverse birth outcomes may lead not only to an increased utilization of immediate postnatal services [10], but also to increased costs to the healthcare system throughout infancy, childhood, and adulthood [11].

Despite large numbers of asylum seekers, no studies have analyzed maternity care services or maternal outcomes among asylum seeking women in Germany between 1994 and 2014 [12]. Updating the search conducted by the most recent systematic review [12] yielded no additional relevant literature in the German context until 31.12.2017.

We here analyze differences in pregnancy outcomes and obstetric services between asylum seeking women living in a large reception center in Germany and resident women, both delivering in a public hospital between 2010 and 2016. We focus on differences between asylum seekers and residents in the prevalence of (i) high-risk pregnancy conditions, (ii) abortive outcomes and stillbirths, (iii) peri- and postnatal maternal complications, and (iv) neonatal complications. Further, we compare obstetric services provided to asylum seekers and residents with respect to caesarean sections and the timing of hospital admission for labor and delivery.

## Methods

### Study context and data sources

We used anonymous, administrative data of the largest hospital (Städtisches Klinikum) in the city of Karlsruhe, Germany, to obtain information on pregnancy outcomes and obstetric services provided to resident women and asylum seeking women from the state reception center Karlsruhe between 2010 and 2016.

Until 2015, the state reception center quasi-randomly received about 13% of newly arriving asylum seekers to Germany based on administrative quota, as it acted as sole reception center for the state of Baden-Württemberg. Further centers were established thereafter in the federal state due to the rising number of asylum seekers. Asylum seekers undergo a mandatory tuberculosis (TB) screening according to §62 of the Asylum Act and remain in the centers until they are transferred to cities in other districts and communities of Baden-Württemberg. In 2015, the maximum duration of stay in the reception centers was prolonged from three to six months, but asylum seekers from so-called “safe” countries of origin remain in the centers throughout the asylum process.

The center provides primary and midwifery care services onsite; specialist outpatient maternity care is provided in private practices. Inpatient specialist services and obstetric care is mainly provided by the public hospital in Karlsruhe. The hospital has a capacity of about 1450 beds and functions as the main tertiary care provider for gynaecological and obstetric services for both resident and asylum seeking women after referral. The only other facility providing obstetric services is a faith-based hospital (with about 530 beds) which is geographically more remote from the reception center facilities.

No standardized medical records exist in the reception center to evaluate antenatal care or determine the total number of pregnant asylum seekers. We hence used data of the mandatory TB screening to approximate the total number of pregnant asylum seekers in the reception center in the period 2010–2016. Pregnant asylum seekers over 15 years old can be identified in the screening data as they undergo a tuberculin skin test or interferon-gamma release assay test, as opposed to the chest radiography administered to non-pregnant individuals.

### Inclusion criteria

We included all women admitted to the gynaecological clinic of the public hospital in Karlsruhe between 01.01.2010 and 31.12.2016 due to conditions related to pregnancy, childbirth, or the post-partum period. Women with any of the codes of chapter XV of the German Modification the International Classification of Diseases (ICD-10-GM version 2017) in their primary or secondary diagnoses were considered eligible for inclusion in the analysis. We excluded women above 50 years of age due to low case numbers among asylum seekers (*n* = 5). A unique cost unit was used to reliably identify and distinguish women with asylum seeker status under state-mandate from resident women covered by statutory sickness funds or private insurance companies.

### Pregnancy outcomes

We used ICD codes to operationalize high-risk pregnancy conditions (ICD-10-GM O10-O16; O20- O29; and O30-O48), abortive outcomes (ICD-10-GM O00-O08) and stillbirths (ICD-10-GM Z37.1; Z37.3; Z37.4; Z37.6; and Z37.7), perinatal maternal complications (ICD-10-GM O60-O75), postnatal maternal complications in the post-partum period (ICD-10-GM O85-O92), and neonatal complications (ICD-10-GM P00-P96). Each outcome was coded as binary (1/0) variable.

### Obstetric services

We used ICD codes and German operation and procedure (OPS) codes to determine the prevalence of caesarean sections (ICD-10-GM O82; O60.1; O60.2; O60.3 or OPS 5–740; 5–741; 5–742; and 5–749). The timing of hospital admission for labor and delivery was coded as variable with four categories according to weeks of gestation:“≤ 25 weeks” (ICD-10-GM O09.0 - O09.3), “26 - 36 weeks” (ICD-10-GM O09.4 - O09.5), “37 - 41 weeks” (ICD-10-GM O09.6), and “> 41 weeks” (ICD O09.7).

### Statistical analysis

We calculated and plotted the prevalence of pregnancy outcomes and caesarian sections per 100,000 women stratified by residence status and age group, as well as the proportion of women admitted to hospital by gestational week. We analyzed differences in pregnancy outcomes and obstetric services between asylum seeking and resident women using single and multiple logistic regression models. All models were adjusted for age, length of admission, and high-risk pregnancy conditions where appropriate. Length of admission was calculated in days using dates of admission and discharge, and the natural logarithm was included in the models. Women’s age was categorized in four groups (12–20, 21–30, 31–40, and 41–50) for descriptive purposes, and included as linear variable in the regression analyses. Regression diagnostics were performed by means of standardized residual plots to rule out heteroscedasticity (data not shown). We calculated attributable fractions among the exposed (*Afe*) to assess the excess rate for selected outcomes among asylum seekers, as well as the attributable fraction of those outcomes among the total population (*Afp*). Stata version 15.1 was used for descriptive analysis, single logistic regression, calculation of *Afe* and *Afp*, and illustration of results. The function glm of the R-statistical package [13] was used for the multiple logistic regression models.

## Results

A total of 42,445 women were admitted to inpatient care of the hospital (2010–2016), of which 2.1% (*n* = 870) were asylum seekers. We excluded 5718 resident women and five asylum seekers aged > 50 years, and 16,562 residents and 296 asylum seekers due to admissions unrelated to pregnancy, childbirth or post-partum complications. The final sample consisted of *N* = 19,864 women of which 2.9% (*n* = 569) were asylum seekers. Data for the timing of hospital admission for labor and delivery was missing for 419 women, 25 of which were asylum seekers; no data was missing for any other variables. The number of asylum seeking women admitted to the hospital during pregnancy, childbirth, or the post-partum period corresponds to 19.3% of all incoming women identified as pregnant (*n* = 2944) in the scope of the mandatory TB screening in the reception center (2010–2016). On average, asylum seeking women were slightly younger than residents and had a shorter length of admission (Table 1).

**Table 1:** Age and length of admission by residence status, *N* = 19,864 women admitted to hospital, 2010–2016

	Resident women		Asylum seeking women		Total population	
	n	%	n	%	N	%
Age group						
12–20	602	3.1	88	15.5	690	3.5
21–30	7417	38.4	314	55.2	7731	38.9
31–40	10,460	54.2	154	27.1	10,614	53.4
41–50	816	4.2	13	2.3	829	4.2
Total	19,295	100.0	569	100.0	19,864	100.0
	M (SD)	Min-Max	M (SD)	Min-Max	M (SD)	Min-Max
Age (years)	31.4 (5.4)	15–50	27.1 (6.2)	14–44	31.3 (5.5)	14–50
Length of admission (days)	4.5 (4.6)	1–97	4.0 (3.1)	1–35	4.5 (4.6)	1–97

*%* column percent, *M* arithmetic mean, *SD* standard deviation, *Min* minimum, *Max* maximum

**Fig. 1:**
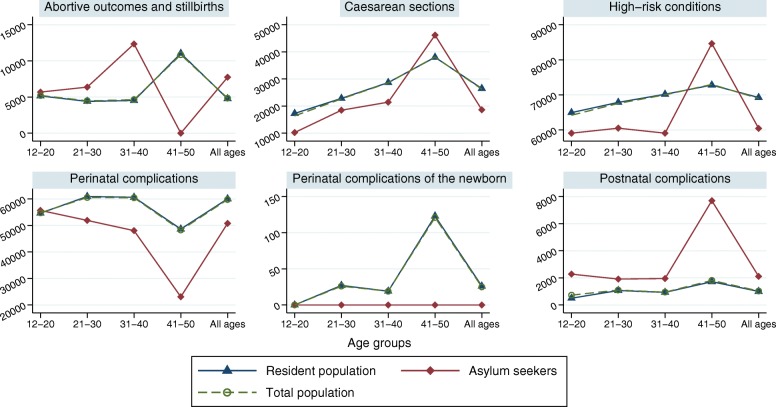
Prevalence of pregnancy outcomes and caesarean sections per 100,000 women by residence status and age group, *N* = 19,864 women admitted to hospital, 2010–2016. Y-axis: Prevalence per 100,000 women

The prevalence (per 100,000) of high-risk pregnancy conditions was lower among asylum seeking women compared to residents, except for the 41–50 age group (Fig. 1). Asylum seeking women had higher prevalence rates (per 100,000) of abortive outcomes and stillbirths (except for the 41–50 age group) and postnatal complications; and lower prevalence rates (per 100,000) of perinatal complications (except for the 12–20 age group). No case of perinatal complications of the newborn was coded among asylum seekers, so that no further analysis was possible for this outcome. Fewer caesarean sections were conducted per 100,000 women among asylum seekers (except for the 41–50 age group).

**Fig. 2:**
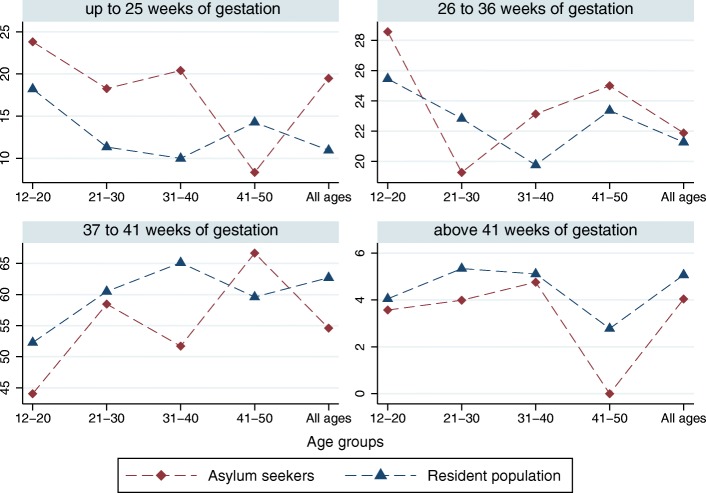
Proportion of women admitted to hospital by week of gestation of pregnancy, residence status, and age group, *N* = 19,445, 2010–2016. Y-axis: Percentage (%) of women

The proportion of women admitted to hospital up to 25 weeks of gestation was higher among asylum seekers compared to residents (except for the 41–50 age group), and lower for admissions above 41 weeks of gestation. The pattern of admissions for other categories of gestational week of pregnancy was varied (Fig. 2). The detailed descriptive data is provided in Additional file 1.

The unadjusted logistic regression analysis showed higher odds of abortive outcomes and stillbirths, postnatal complications, and admissions up to 25 weeks of gestation among asylum seekers relative to resident women (Table 2).

**Table 2:** Crude odds ratio of pregnancy outcomes and obstetric services for asylum seekers relative to resident women, *N* = 19,864 women admitted to hospital, 2010–2016

	Crude Odds Ratio (Ref.: resident women)	95% CI	*p*-value
Pregnancy outcome			
High-risk pregnancy	**0.68**	**(0.57–0.81)**	**< 0.0001**
Abortive outcomes/stillbirths	**1.68**	**(1.22–2.30)**	**0.001**
Perinatal complications	**0.69**	**(0.58–0.81)**	**< 0.0001**
Postnatal complications	**2.13**	**(1.18–3.84)**	**0.012**
Obstetric care services			
Caesarean sections	**0.64**	**(0.51–0.79)**	**< 0.0001**
Timing of hospital admission^a^			
Up to 25 weeks of gestation	**1.97**	**(1.59–2.45)**	**< 0.0001**
26–36 weeks of gestation	1.04	(0.84–1.27)	0.736
37–41 weeks of gestation	**0.71**	**(0.60–0.85)**	**< 0.0001**
> 41 weeks of gestation	0.79	(0.51–1.22)	0.283

*Ref* Reference category, *CI* confidence interval. ^a^*N* = 19,445 women (*n* = 18,901 residents and *n* = 544 asylum seekers). Figures in boldface: statistically significant below the 0.05 level

The excess rate of abortive outcomes and stillbirths among asylum seekers (*Afe*) was 40.3% (95% confidence interval, CI: 16.3–56.5). These outcomes correspond to 1.8% of abortive outcomes and stillbirths in the total population of women (*Afp*). For postnatal complications, the excess rate (*Afe)* among asylum seekers was 53.1% (95% CI: 7.1–74.0) and the *Afp* was 3.1%.

The unadjusted odds of high-risk pregnancy conditions, perinatal maternal complications, caesarean sections, and admissions during 37 to 41 gestational weeks of pregnancy were significantly (*p* < 0.05) lower among asylum seekers compared to residents (Table 2).

**Fig. 3:**
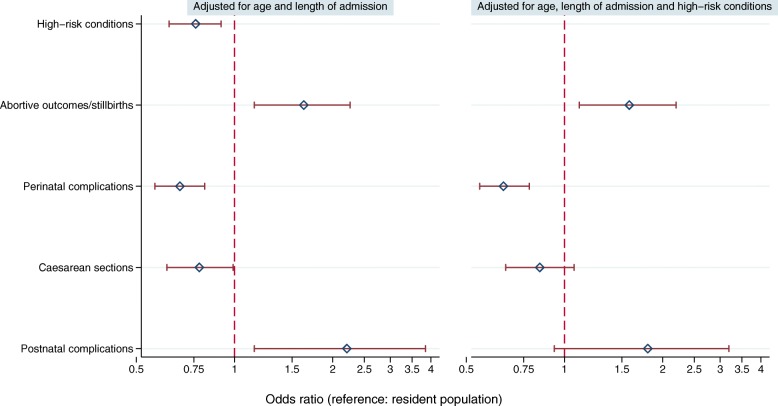
Adjusted odds ratios of pregnancy outcomes and obstetric services for asylum seekers relative to resident women, *N* = 19,864 women admitted to hospital, 2010–2016. Y-axis: logarithmic scale

These patterns were confirmed by the multiple regression analysis: the direction of the associations remained stable in the adjusted models, but the strength of the association was slightly reduced (Fig. 3). Differences in caesarean sections and postnatal complications between asylum seeking women and residents were slightly attenuated after full adjustment for age and morbidity variables (Fig. 3). A detailed overview of regression estimates including those of co-variables in the adjusted models is provided in Additional file 2.

## Discussion

This is the first study in Germany quantifying differences in pregnancy outcomes and obstetric services between asylum seeking women in a state reception center and resident women. Asylum seeking women were at considerably higher risk for abortive outcomes and stillbirths compared to resident women after full adjustment for age and morbidity-related variables. The excess rate of abortive outcomes and stillbirths among asylum seekers was large. Reducing the rate of adverse outcomes in this group to a level observed among resident women would thus entail high health gains. Several factors across the migration trajectory, ranging from pre- and peri-migration factors to the conditions in the destination country can be associated with the high level of abortions and stillbirths. The lack of adequate health services in the country of origin, perilous migration journeys and near-death experiences, as well as the living conditions in reception centers are experienced as very stressful for asylum seeking women in Germany, affecting their health-related quality of life [14]. Frequent dispersals before reaching the final reception center may also contribute to adverse outcomes and interrupt the continuity of care leading to severe consequences for health and well-being of pregnant women seeking asylum [15, 16]. Furthermore, untreated mental conditions such as depression, anxiety disorders or traumatic stress have adverse consequences for women and their (unborn) children [17]. As shown in a systematic review and meta-analysis in migrant women from low- and middle-income countries, the prevalence of mental illness during pregnancy is very high (e.g. 31.4% for any depressive disorder and 17.3% for major depression) [17]. Adequate and timely identification of mental illness during pregnancy by means of standardized and routinely applied screening interventions, as well as provision of low-threshold psychosocial support based on individual need are thus needed. Such approaches and care provision models, however, do currently not exist in Germany. Integrating routine screening and psychosocial support into maternity care services in reception centers would also be in line with binding EU directives [1] requesting member states to establish process for identifying and addressing the special needs of vulnerable groups among asylum seekers.

Our study further identified the post-partum period as particularly important to improve maternity care for asylum seeking women: the adjusted odds of admission due to conditions related to the post-partum period was considerably higher compared to residents. We found no indication of delayed referrals using the proxy indicator of gestational weeks of pregnancy to assess differences in timing of hospital admission for labor and delivery. Other adverse outcomes such as perinatal maternal and neonatal complications were lower among asylum seeking women. This could either be related to a higher parity or - in light of the lower odds for high-risk pregnancy conditions - indicate a healthy migrant effect. A health advantage of recent immigrants compared to the native population with comparable socio-demographic characteristics has been observed in several studies [18], also in the context of maternity care and pregnancy outcomes among regular immigrants in Germany [19]. This phenomenon is usually attributed to a self-selection prior to migration, and used to explain why recent immigrants perform better on some health measures despite lower socio-economic status, adverse living conditions, and various barriers to health care [18].

This study has important strengths and limitations. Using administrative hospital data, we reliably identified a group of vulnerable asylum seekers living in reception centers. This allowed us to quantify differences in relevant clinical outcomes related to pregnancy, childbirth, and post-partum and compare patterns between asylum seeking and resident women over a period of 6 years. We estimate that a large proportion (19.3%) of pregnant asylum seekers were covered by the hospital dataset, but this estimation needs to be interpreted with care. Women becoming pregnant after the health examination while still living in the reception center and pregnant women under 15 years old are not captured using the screening data approach. The unique geographical setting of the public hospital in Karlsruhe as the main referral facility for the reception center minimized - but did not rule out - a referral bias inherent in any study using hospital-based data. The study findings are valid for pregnant asylum seeking women in reception centers, but may not be transferable to asylum seekers who have been transferred to districts and communities. Although the transfer of pregnant asylum seeking women is purely based on administrative criteria, it may not be ruled out that protective factors (e.g. having a more private accommodation) or risk factors (e.g. high number of transfers and discontinuity of care) exert different effects of pregnancy outcomes and access to maternity care.

The use of routine data entailed a lack of relevant socio-demographic parameters (such as country of origin, spoken language, religion or maternal education) and clinical parameters (such as gravidity and parity), so that residual confounding cannot be ruled out. We also lacked information on antenatal care services provided to women, so that the timing of admission is only a crude approximation of the timeliness of outpatient care provision. Better parameters such as early antenatal coverage are needed to monitor equity in access to maternity services [20] for this vulnerable population group. Data on cost of care is theoretically available in the accounts data used here, but the data was not practically available at time of conducting the study. Future research should address this issue and compare morbidity-adjusted differences in cost of care between asylum seekers and residents, making economic evidence available to inform policy and practice and add to the scarce evidence base [10] in this area.

Our findings on elevated risk of stillbirths among asylum seekers are consistent with numerous studies in other countries conducted among general migrant or refugee populations [6–8]. Using the German perinatal data base, Reeske et al. reported an increased risk of stillbirths in migrants (without reference to their residence status) originating from the Middle East and North Africa [21]. A study in Canada reported higher clinical and psychosocial health needs in asylum seeking and refugee women in the postnatal period [22]. The prevalence of caesarean sections (per 100,000) in both residents and asylum seekers was lower than the aggregate national average of 30,700 [23] indicating regional variations. While there is mixed evidence on caesarean section rates among migrants [6–8], a case-control study in London found no significant differences between refugees from Kosovo and residents in the rates of caesarean sections [24]. Overall, cross-country comparisons of such results are very difficult due to differences in societal and health system factors, as well as differences between the underlying migrant populations studied.

The findings of this study have important implications for clinicians and policymakers: pregnant asylum seekers in reception centers are at higher risk of abortive outcomes and stillbirths despite a lower morbidity as measured by high-risk pregnancy conditions. The relative lack of systematic data collection on this population renders such health inequalities invisible. A higher risk of abortive outcomes and stillbirths in asylum seeking women that goes unnoticed by the health system can be regarded as an uncounted toll of forced migration. A wide range of individual circumstances (such as socio-economic status), but also adverse experiences before and during flight [14] can be associated with such negative birth outcomes. However, the post-migration phase is a critical period in which individual risk factors and prior experiences can be mitigated or exacerbated.

Ensuring safe and private accommodation, food security, good access to full antenatal and preventive services, and access to psychosocial and specialized mental health services may help to mitigate negative experiences of the pre- and peri-migration phase. The tendency towards higher risk of postnatal complications in asylum seekers requires improvements in outpatient care in the post-partum period, including better follow-up and continuity of care. Scaling up midwifery care in the post-partum period and strengthening the outreach capacity of maternity care teams may be crucial to achieve this. Maternity care teams, consisting of midwives, social workers, and psychologists implementing care pathways for at-risk women may make big differences in health outcomes for asylum seeking pregnant women [15].

Further research is, however, needed to better understand and explain the reasons for the higher levels of adverse outcomes such as abortions and postnatal complications among asylum seekers. Such research should consider the role of post-migration factors and the relative contribution of pre- and peri-migration factors to adverse birth outcomes among asylum seekers. To this end, prospective studies with expanded geographical reach and better linkage of data from reception centers with data of ante- and perinatal care services are needed.

## Conclusions

This study offers first insights into pregnancy outcomes and obstetric care among asylum seeking women using data from a reception center of the third largest federal state in Germany over a period of 7 years. Despite the fact that high-risk pregnancy conditions are lower among asylum seeking compared to resident women, we find substantially higher adjusted odds for postnatal maternal complications and abortive outcomes/stillbirths among asylum seekers. Other adverse outcomes such as perinatal maternal and neonatal complications are lower among asylum seeking women compared to resident women. Measures to improve outpatient postnatal care in reception centers and effectively mitigate adverse birth outcomes among asylum seeking women are needed. Although further research on modifiable post-migration risk factors for such outcomes is needed, scale up of midwifery care in reception centers, improved outreach by maternity care services, and routine identification of mental illness and psychosocial care during pregnancy could be ways forward to improve pregnancy outcomes in this population.

## Additional files

Additional file 1: Absolute frequency and prevalence (per 100,000) of pregnancy outcomes and caesarean sections by age group and residence status, *N* = 19,864 women. (PDF 77 kb)

Additional file 2: Regression coefficients and odds ratios with 95% confidence intervals for multivariate logistic regression models. (PDF 96 kb)

## Abbreviations

Afe: Attributable fractions among the exposed
Afp: Attributable fractions among the total population
CI: Confidence interval
ICD-10-GM: German Modification of the International Classification of Disease
OPS: German operation and procedure codes
OR: Odds ratio
TB: tuberculosis
