# Supplementary material for: Differences in pregnancy outcomes and obstetric care between asylum seeking and resident women: a cross-sectional study in a German federal state, 2010–2016
Source: BMC Pregnancy Childbirth. 2018 Oct 24;18:417. doi: 10.1186/s12884-018-2053-1 (PMC6201533; doi:10.1186/s12884-018-2053-1)
Supplement: Supplementary file 1 — Absolute frequency and prevalence (per 100,000) of pregnancy outcomes and caesarean sections by age group and residence status, N = 19,864 women. (PDF 77 kb) [file 12884_2018_2053_MOESM1_ESM.pdf]

Additional file 1: Absolute frequency and prevalence (per 100,000) of pregnancy outcomes and caesarean sections by age group and residence status, N=19,864 women

	Age group	Resident women		Asylum seekerum seeking women		Total	
		n	%	n	%	N	%
	12-20	602	3.1	88	15.5	690	3.5
	21-30	7,417	38.4	314	55.2	7,731	38.9
	31-40	10,460	54.2	154	27.1	10,614	53.4
	41-50	816	4.2	13	2.3	829	4.2
	all	19,295	100.0	569	100.0	19,864	100.0
**Parameter**							
		**n**	**Prevalence per 100,000**	**n**	**Prevalence per 100,000**	**N**	**Prevalence per 100,000**
High-risk pregnancies pregnancy conditions	12-20	391	64,950	52	59,091	443	64,203
	21-30	5,034	67,871	190	60,510	5,224	67,572
	31-40	7,341	70,182	91	59,091	7,432	70,021
	41-50	594	72,794	11	84,615	605	72,979
	all	13,360	69,241	344	60,457	13,704	68,989
Abortive outcomes and stillbirths	12-20	31	5,150	5	5,682	36	5,217
	21-30	326	4,395	20	6,369	346	4,475
	31-40	472	4,512	19	12,338	491	4,626
	41-50	90	11,029	0	0	90	10,856
	all	919	4,763	44	7,733	963	4,848
Perinatal complications	12-20	329	54,651	49	55,682	378	54,783
	21-30	4,519	60,928	163	51,911	4,682	60,561
	31-40	6,343	60,641	74	48,052	6,417	60,458
	41-50	397	48,652	3	23,077	400	48,251
	all	11,588	60,057	289	50,791	11,877	59,792
Caesarean sections	12-20	104	17,276	9	10,227	113	16,377
	21-30	1,693	22,826	58	18,471	1,751	22,649
	31-40	2,998	28,662	33	21,429	3,031	28,557
	41-50	310	37,990	6	46,154	316	38,118
	all	5,105	26,458	106	18,629	5,211	26,233
Postnatal complications in post-partum period	12-20	3	498	2	2,273	5	725
	21-30	79	1,065	6	1,911	85	1,099
	31-40	97	927	3	1,948	100	942
	41-50	14	1,716	1	7,692	15	1,809
	all	193	1,000	12	2,109	205	1,032
Perinatal complications of the newborn	12-20	0	0	0	0	0	0
	21-30	2	27	0	0	2	26
	31-40	2	19	0	0	2	19
	41-50	1	123	0	0	1	121
	all	5	26	0	0	5	25
