# Supplementary material for: Differences in pregnancy outcomes and obstetric care between asylum seeking and resident women: a cross-sectional study in a German federal state, 2010–2016
Source: BMC Pregnancy Childbirth. 2018 Oct 24;18:417. doi: 10.1186/s12884-018-2053-1 (PMC6201533; doi:10.1186/s12884-018-2053-1)
Supplement: Supplementary file 2 — Regression coefficients and odds ratios with 95% confidence intervals for multivariate logistic regression models. (PDF 96 kb) [file 12884_2018_2053_MOESM2_ESM.pdf]

Additional file 2: Regression coefficients and odds ratios with 95 % confidence intervals for multivariate logistic regression models

Modelnr.	Outcome	Independent Variables	β	2.5%	97.5%	Odds Ratio	2.5%.	97.5%.	p-value
1	High-High-risk pregnancies pregnancy conditions	Asylum seeker	-0.28	-0.46	-0.10	0.76	0.63	0.91	0.00
		Age (years)	0.01	0.00	0.01	1.01	1.00	1.01	0.01
		log(dur)	0.87	0.82	0.93	2.40	2.27	2.53	0.00
2	Abortive outcomes/stillbirths	Asylum seeker	0.49	0.14	0.81	1.63	1.15	2.26	0.00
		Age (years)	0.05	0.03	0.06	1.05	1.04	1.06	0.00
		log(dur)	-1.56	-1.67	-1.46	0.21	0.19	0.23	0.00
3	Perinatal complications	Asylum seeker	-0.38	-0.56	-0.21	0.68	0.57	0.81	0.00
		Age (years)	-0.02	-0.02	-0.01	0.98	0.98	0.99	0.00
		log(dur)	0.86	0.81	0.91	2.37	2.25	2.49	0.00
4	Caesarean Sections	Asylum seeker	-0.25	-0.48	-0.01	0.78	0.62	0.99	0.04
		Age (years)	0.04	0.03	0.05	1.04	1.03	1.05	0.00
		log(dur)	1.77	1.69	1.84	5.85	5.44	6.30	0.00
5	Postnatal complications	Asylum seeker	0.79	0.14	1.35	2.21	1.15	3.85	0.01
		Age (years)	0.01	-0.02	0.03	1.01	0.98	1.03	0.64
		log(dur)	0.08	-0.13	0.29	1.09	0.88	1.34	0.44
6	Perinatal neonatal complication	Asylum seeker	-13.84		99.81	0.00		9.9e-01	0.99
		Age (years)	0.12	-0.04	0.30	1.13	0.96	1.35	0.15
		log(dur)	-0.31	-1.64	0.99	0.74	0.19	2.69	0.66
7	Abortive outcomes/stillbirths	Asylum seeker	0.46	0.11	0.79	1.58	1.11	2.20	0.01
		Age (years)	0.05	0.04	0.06	1.05	1.04	1.06	0.00
		log(dur)	-1.47	-1.58	-1.36	0.23	0.21	0.26	0.00
		HIGH-RISK PREGNANCIES	-1.17	-1.31	-1.03	0.31	0.27	0.36	0.00
8	Perinatal complicatons	Asylum seeker	-0.43	-0.61	-0.25	0.65	0.55	0.78	0.00
		Age (years)	-0.02	-0.02	-0.01	0.98	0.98	0.99	0.00
		log(dur)	0.98	0.93	1.03	2.66	2.52	2.81	0.00
		HIGH-RISK PREGNANCIES	-0.60	-0.67	-0.53	0.55	0.51	0.59	0.00
9	Caesarean Sections	Asylum seeker	-0.17	-0.41	0.07	0.84	0.66	1.07	0.17
		Age (years)	0.04	0.03	0.05	1.04	1.03	1.05	0.00
		log(dur)	1.57	1.50	1.64	4.80	4.46	5.17	0.00
		HIGH-RISK PREGNANCIES	1.13	1.03	1.22	3.09	2.81	3.40	0.00
10	Postnatal complications	Asylum seeker	0.59	-0.07	1.16	1.80	0.93	3.19	0.06
		Age (years)	0.01	-0.01	0.04	1.01	0.99	1.04	0.35
		log(dur)	0.97	0.69	1.25	2.64	2.00	3.50	0.00
		HIGH-RISK PREGNANCIES	-4.35	-5.12	-3.72	0.01	0.01	0.02	0.00

*log(dur): natural logarithm of duration of admission.
